# Supplementary figures and images for: High-Fiber, Whole-Food Dietary Intervention Alters the Human Gut Microbiome but Not Fecal Short-Chain Fatty Acids
Source: mSystems. 2021 Mar 16;6(2):e00115-21. doi: 10.1128/mSystems.00115-21 (PMC8546969; doi:10.1128/mSystems.00115-21)

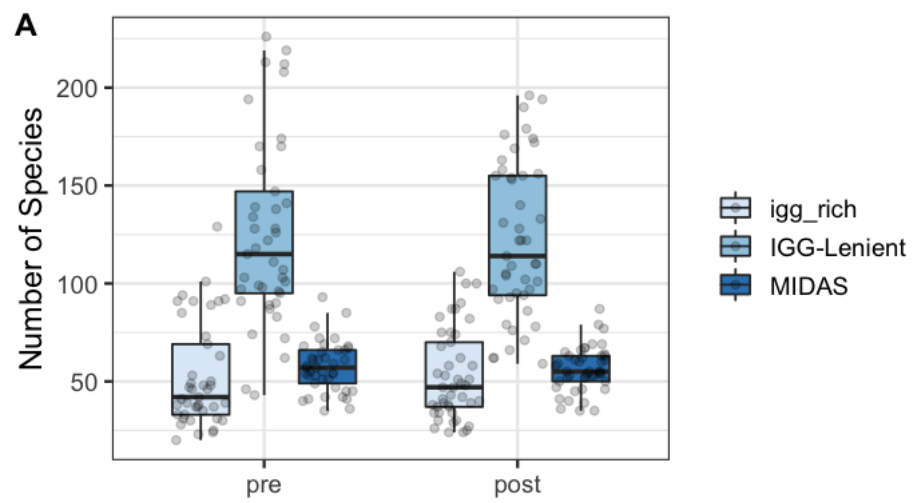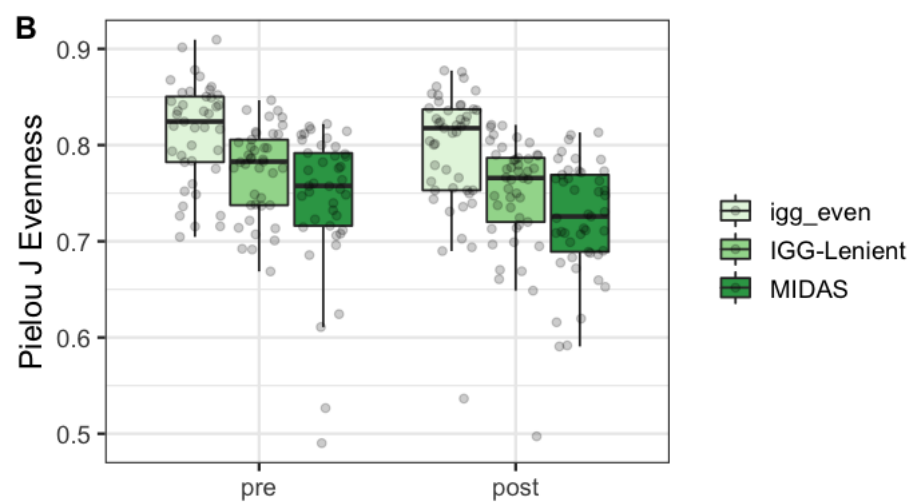

Supplement: FIG S1 [file msystems.00115-21-sf001.pdf]

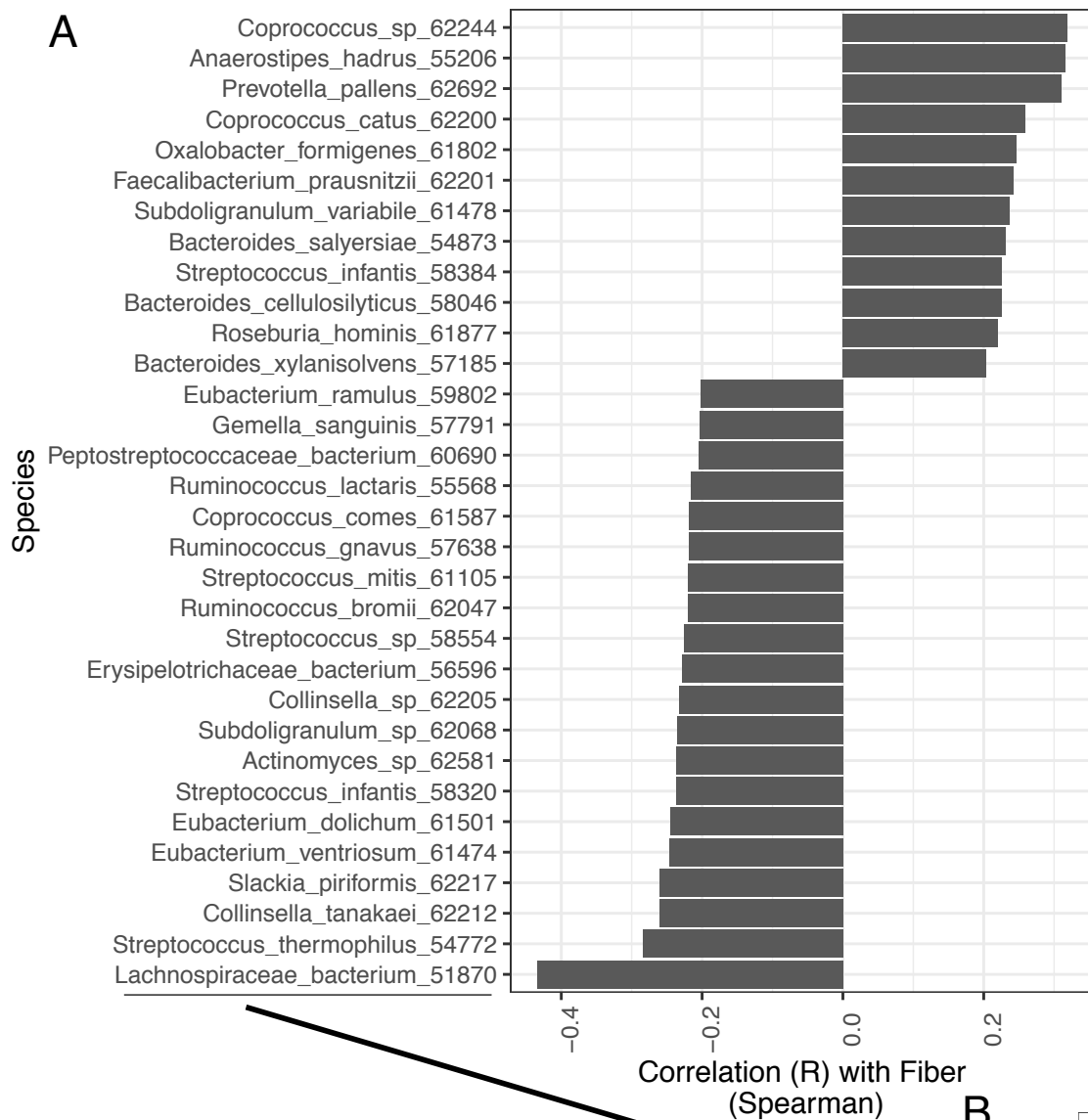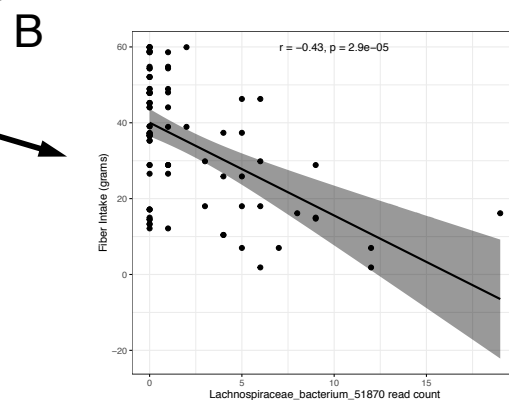

Supplement: FIG S2 [file msystems.00115-21-sf002.pdf]

A

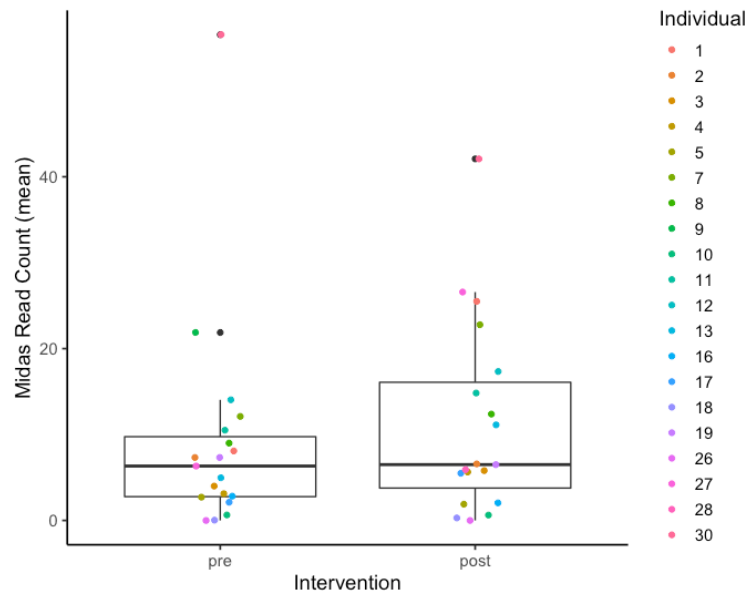

B

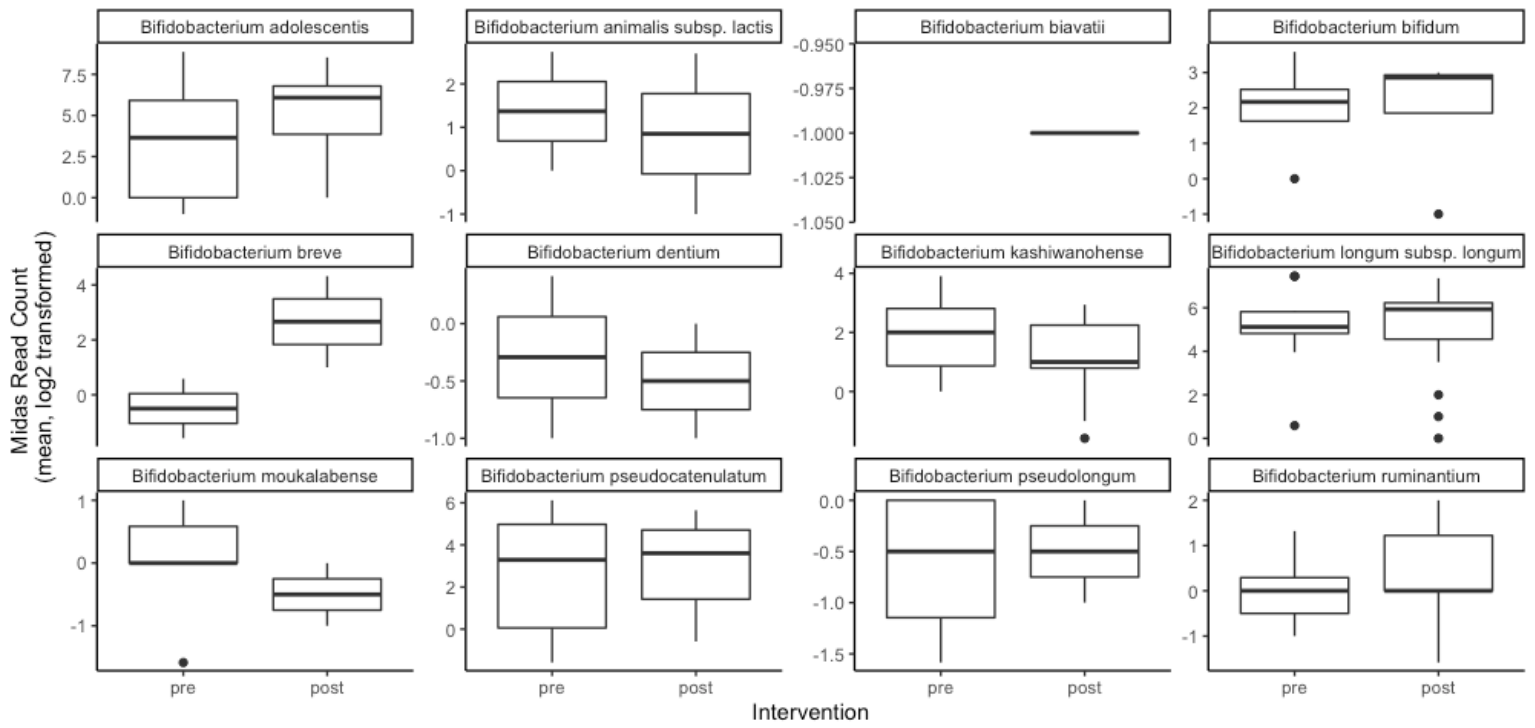

Supplement: FIG S3 [file msystems.00115-21-sf003.pdf]

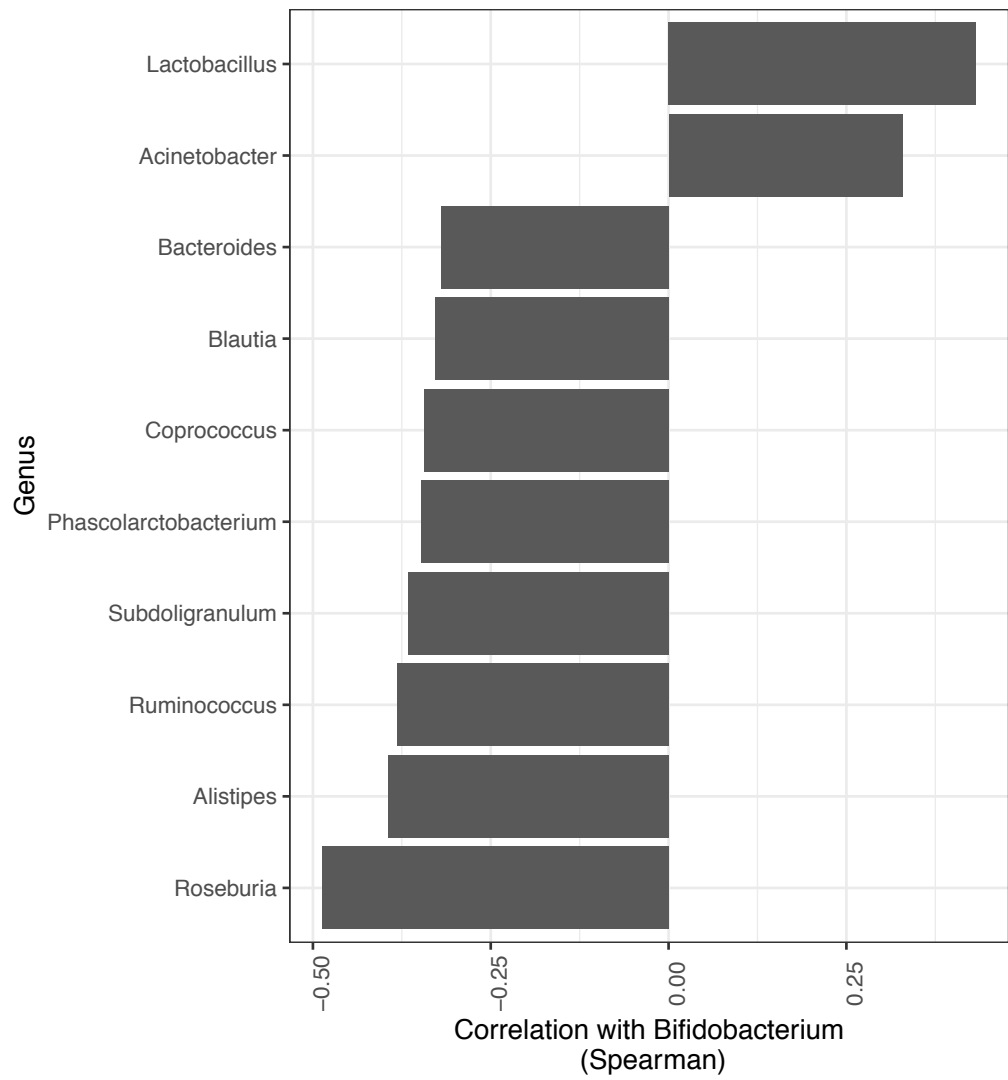

Supplement: FIG S5 [file msystems.00115-21-sf005.pdf]

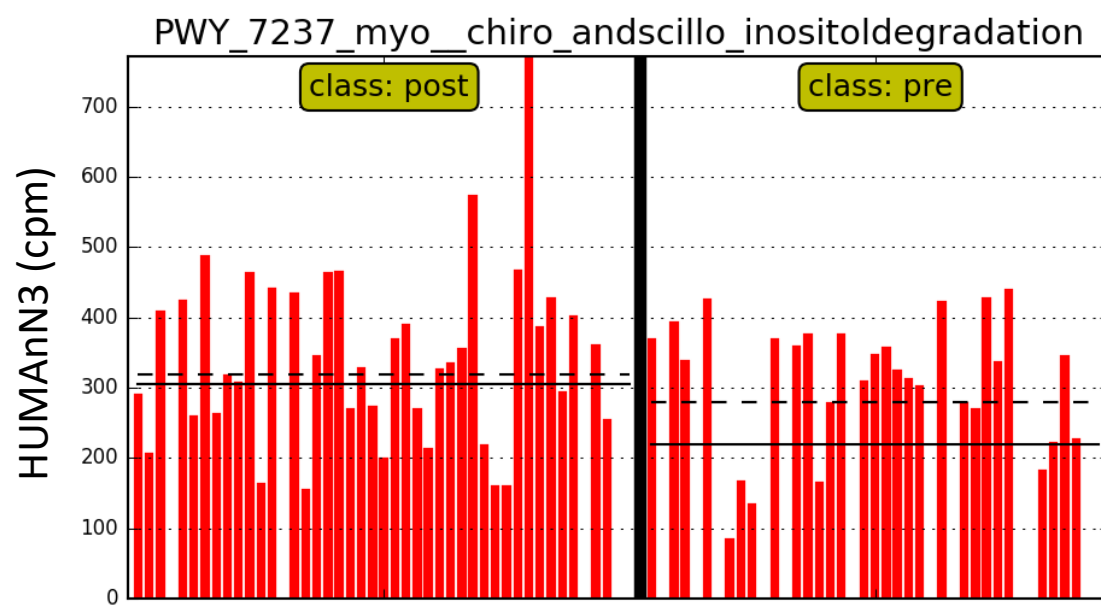

Supplement: FIG S7 [file msystems.00115-21-sf007.pdf]

A

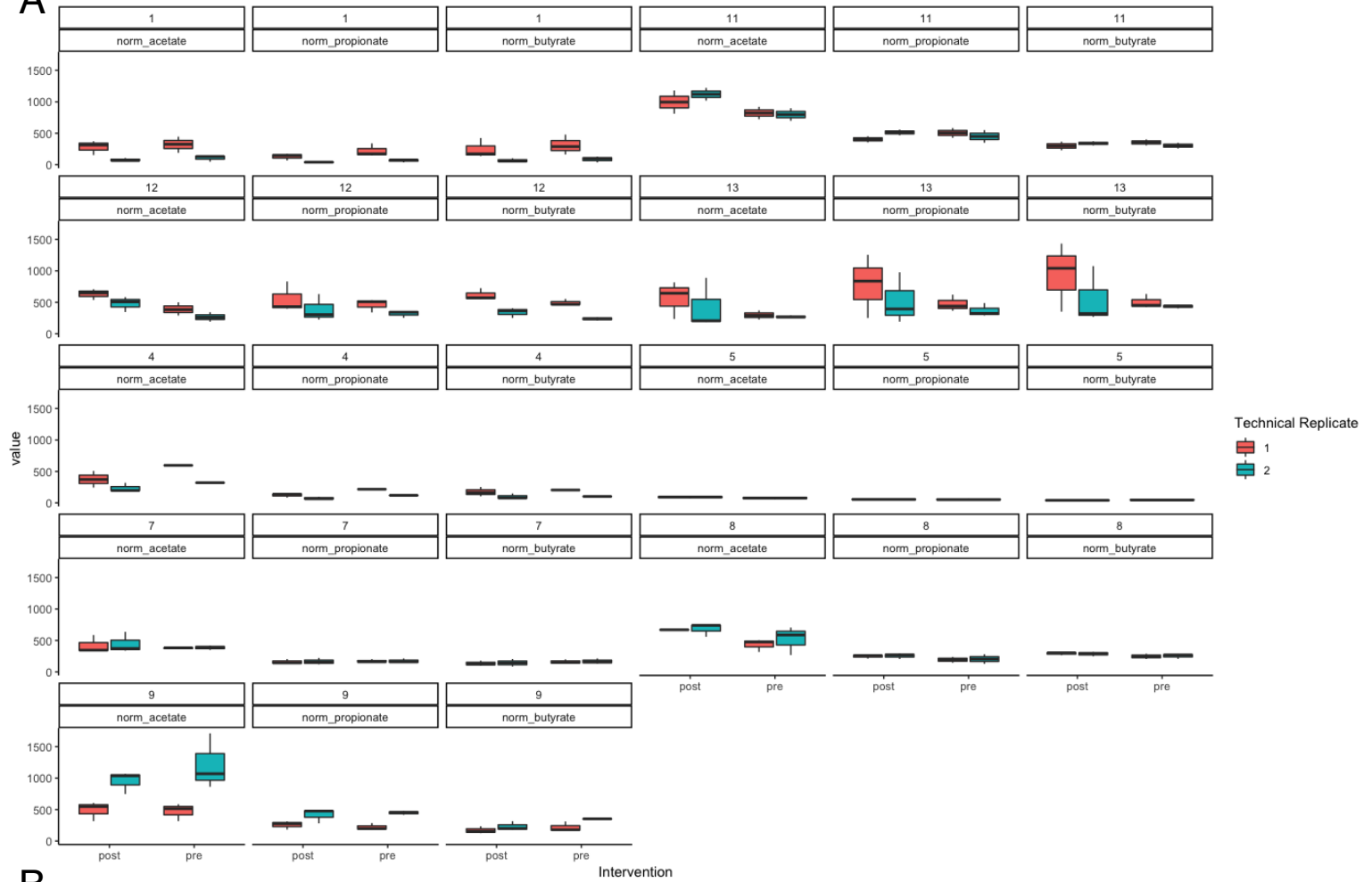

B

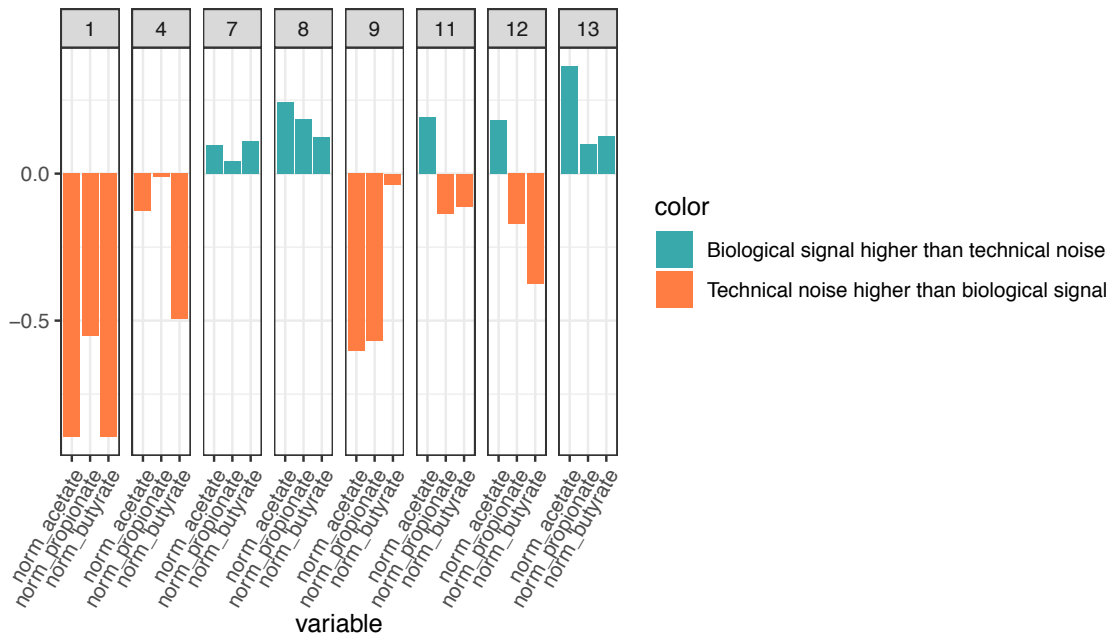

Supplement: FIG S8 [file msystems.00115-21-sf008.pdf]
