# Supplementary material for: High-Fiber, Whole-Food Dietary Intervention Alters the Human Gut Microbiome but Not Fecal Short-Chain Fatty Acids
Source: mSystems. 2021 Mar 16;6(2):e00115-21. doi: 10.1128/mSystems.00115-21 (PMC8546969; doi:10.1128/mSystems.00115-21)
Supplement: TABLE S2 [file msystems.00115-21-st002.docx]

|  | **Df** | **SS** | **MS** | **F.Model** | **R2** | **Pr(>F)** |  |  |
| --- | --- | --- | --- | --- | --- | --- | --- | --- |
| **Individual** | 19 | 12.6885 | 0.66782 | 16.6248 | 0.78397 | 0.001 | *** |  |
| **Intervention** | 1 | 0.2233 | 0.22334 | 5.5598 | 0.0138 | 0.001 | *** |  |
| **Individual : Intervention** | 17 | 1.3449 | 0.07911 | 1.9694 | 0.0831 | 0.001 | *** |  |
| **Residuals** | 48 | 1.9282 | 0.04017 | 0.11913 |  |  |  |  |
| **Total** | **85** | **16.1849** | **1** |  |  |  |  |  |
|  |  |  |  |  |  |  |  |  |
| Model: (community_matrix ~ Individual*Intervention, permutations = 999, method = "bray") | | | | | | |  |  |
