# Supplementary material for: High-Fiber, Whole-Food Dietary Intervention Alters the Human Gut Microbiome but Not Fecal Short-Chain Fatty Acids
Source: mSystems. 2021 Mar 16;6(2):e00115-21. doi: 10.1128/mSystems.00115-21 (PMC8546969; doi:10.1128/mSystems.00115-21)
Supplement: FIG S4 [file msystems.00115-21-sf004.pdf]

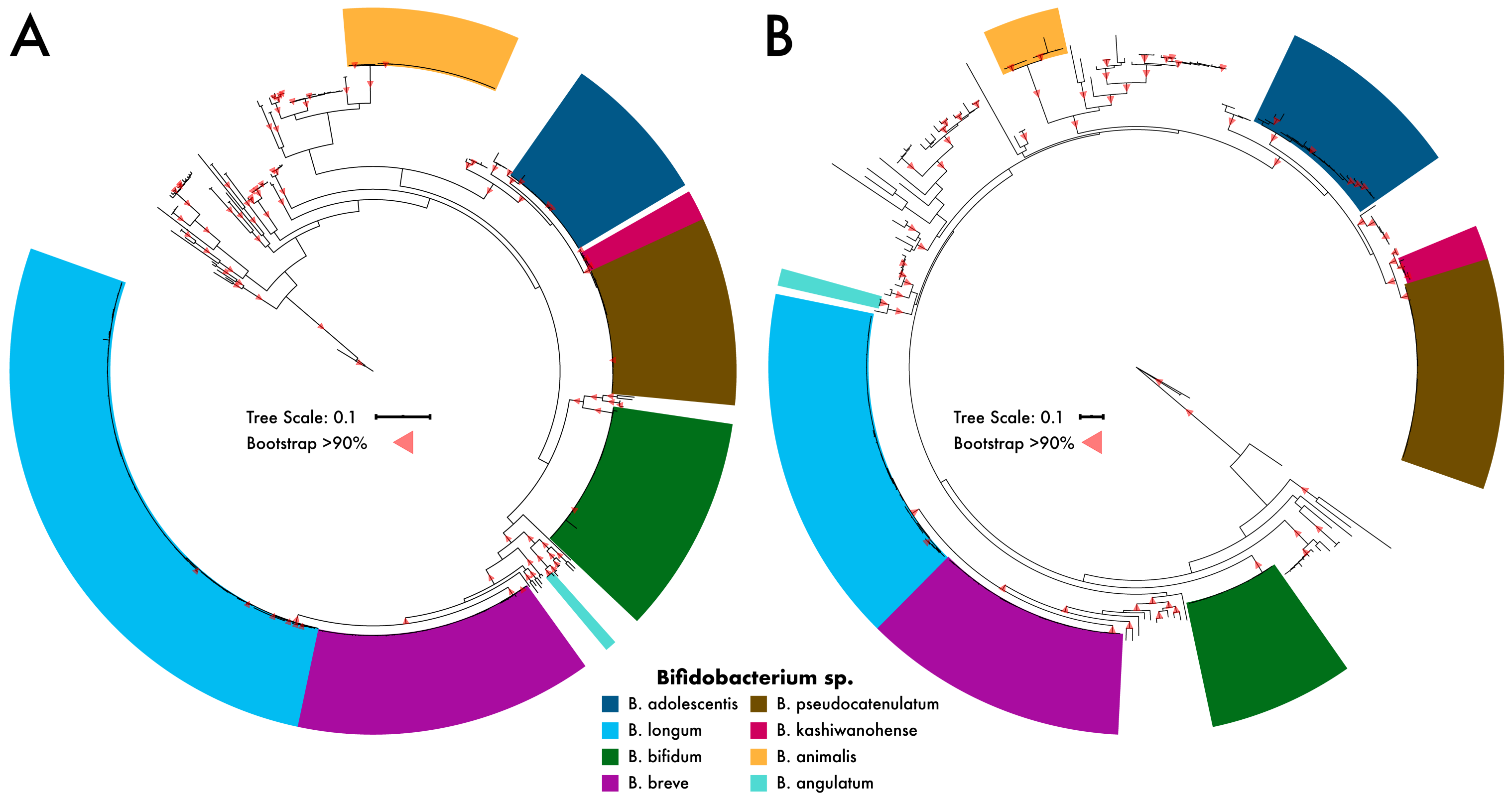
A
B
Tree Scale: 0.1
Bootstrap >90%
Tree Scale: 0.1
Bootstrap >90%
Bifidobacterium sp.
B. adolescentis
B. longum
B. bifidum
B. breve
B. pseudocatenulatum
B. kashiwanohense
B. animalis
B. angulatum
