# Supplementary material for: High-Fiber, Whole-Food Dietary Intervention Alters the Human Gut Microbiome but Not Fecal Short-Chain Fatty Acids
Source: mSystems. 2021 Mar 16;6(2):e00115-21. doi: 10.1128/mSystems.00115-21 (PMC8546969; doi:10.1128/mSystems.00115-21)

A

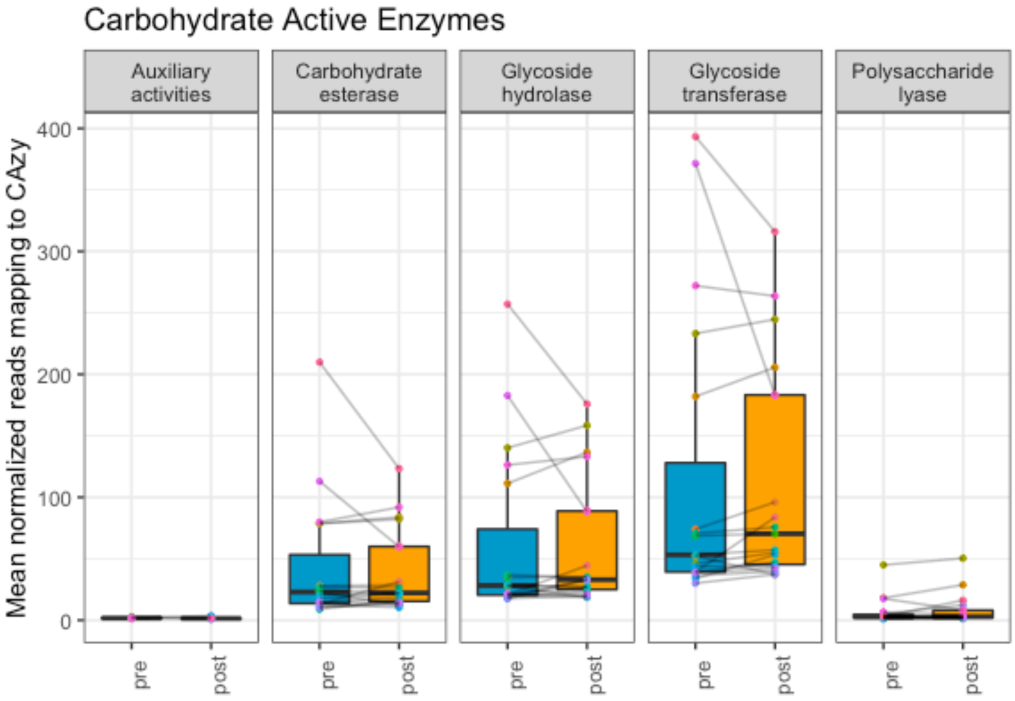

Enzyme_Class	read_count	distinct_enzymes	distinct_Families
Auxiliary activities	48	5	5
Carbohydrate esterase	91406	46	46
Glycoside hydrolase	670165	185	109
Glycoside transferase	187298	39	39
Polysaccharide lyase	11335	28	15

B

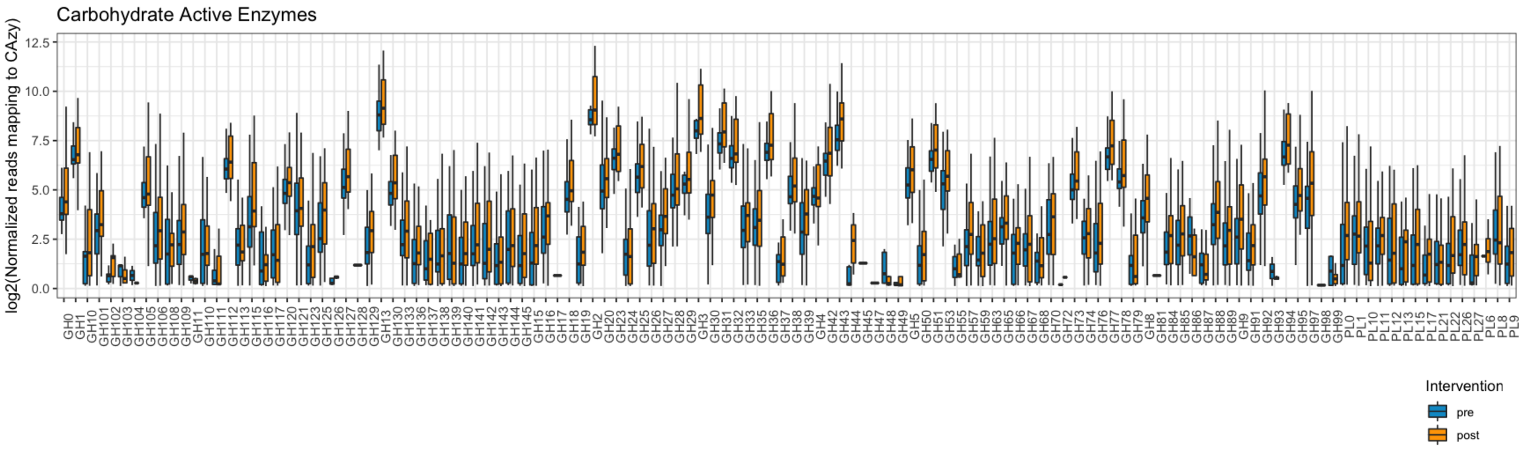

Supplement: FIG S6 [file msystems.00115-21-sf006.pdf]
